# Effects of Probiotics as an Adjunct to Non-Surgical Periodontal Therapy (NSPT): A Narrative Review

**DOI:** 10.3390/jcm14145114

**Published:** 2025-07-18

**Authors:** Leopoldo Mauriello, Alessandro Cuozzo, Vitolante Pezzella, Vincenzo Iorio-Siciliano, Gaetano Isola, Gianrico Spagnuolo, Luca Ramaglia, Andrea Blasi

**Affiliations:** 1 School of Dental Medicine, University of Naples Federico II, Via S. Pansini 5, 80131 Naples, Italy; leopmau96@gmail.com (L.M.); alessandro.cuozzo@unina.it (A.C.); enzois@libero.it (V.I.-S.); gspagnuo@unina.it (G.S.); luca.ramaglia@unina.it (L.R.); andrea.blasi@unina.it (A.B.); 2 Unit of Periodontology, Department of General Surgery and Medical-Surgical Specialities, University of Catania, 95124 Catania, Italy; gaetano.isola@unict.it; 3 Therapeutic Dentistry Department, Institute for Dentistry, Sechenov University, 119991 Moscow, Russia

**Keywords:** non-surgical periodontal therapy, adjunctive therapy, periodontal pockets, probiotics, periodontal disease, *Lactobacillus*

## Abstract

**Background:** Periodontitis is a chronic inflammatory disease characterized by the destruction of periodontal tissues due to biofilm deposits and altered host immune response. Non-surgical periodontal therapy (NSPT) still represents the gold standard for treatment; however, its effectiveness in deep periodontal pockets is limited. Probiotics seem to modulate both oral microbiota and inflammatory response and have been proposed as an adjunctive therapy to NSPT. **Methods**: An electronic search was conducted on PubMed, Scopus, Medline, and Google Scholar to identify English randomized controlled trials (RCTs) evaluating probiotics as adjunctive treatments to NSPT. Studies were selected based on inclusion and exclusion criteria, and clinical outcomes such as probing depth (PD) and clinical attachment level (CAL) were extracted and analyzed. **Results**: Seven RCTs met the inclusion criteria. These studies showed significant improvements in PD and CAL when probiotics were used, specifically with *Lactobacillus* and *Bifidobacterium* species. However, outcomes ranged depending on the strain, dosage, and delivery method. **Conclusions**: Probiotics may be used as an adjunct treatment to NSPT; however, further studies are needed to define valid clinical guidelines.

## 1. Introduction

Periodontitis is an inflammatory chronic disease characterized by a progressive loss of periodontal supportive tissues. Clinically it manifests with gingival inflammation, bleeding, the presence of periodontal pockets, recession, and tooth mobility and migration [1]. Periodontitis is caused by bacterial biofilm together with an altered immune host response [2,3]. The endpoint of periodontal therapy is to arrest disease progression. Clinical success is reached when a periodontal probing depth (PD) ≤ 4 mm with negative bleeding on probing (BoP) is recorded [1]. The aforementioned results are, in fact, most likely to be maintained over time, preventing periodontitis recurrence [4]. Conversely, the presence of deep periodontal pockets (PD ≥ 6 mm) and furcation involvements represents a risk factor for tooth loss and disease progression [5,6]. The European Federation of Periodontology (EFP)’s most recent guidelines propose a stepwise approach: the first two steps of therapy are based on risk factors control, oral hygiene instructions, the patient’s motivation, and both supra and subgingival debridement. Step III is mainly focused on the surgical treatment of non-responsive sites (PD ≥ 4 mm; positive BoP), while the last step of periodontal therapy is supportive periodontal care (SPC) [1]. Thus, the first line of treatment for periodontitis is non-surgical periodontal therapy (NSPT), which still represents the gold standard [7]. NSPT, however, has its limitations, as a mean PD reduction of 1.4 mm has been recorded at 6 months re-evaluation [8], meaning that in deep periodontal pockets a surgical intervention may be necessary in order to achieve clinical success, which increases both the patient’s cost and discomfort [9]. Hence, to avoid surgery and improve the effects of NSPT, new strategies have been proposed, in particular for the treatment of intrabony defects. For instance, the minimally invasive non-surgical technique (MINST) was introduced, and shows promising results [10,11,12]. To further increase the efficacy of NSPT, different molecules have been proposed as adjunctive therapy to improve the clinical outcomes of steps I and II of periodontal therapy, such as antiseptic, systemic, and locally delivered antibiotics [13,14], hyaluronic acid [15], and probiotics [16].

Probiotics are a heterogeneous group of live microorganisms that confer health benefits when administered in adequate amounts. They are able to compete with pathogens and modulate host immune responses, making them ideal candidates for enhancing periodontal outcomes. Probiotics can inhibit pathogens like *P. gingivalis*, modulate biofilm composition, reduce inflammatory cytokines, and promote tissue homeostasis [17].

Probiotic species such as *Lactobacillus reuteri*, *L. rhamnosus*, and *L. salivarius* showed interesting effects as an adjunctive therapy to NSPT, improving several clinical parameters. In fact, they seem to reduce periodontal probing depth (PPD), bleeding on probing (BoP), and the plaque index (PI). In moderate pockets (4–6 mm) statistically significant PPD reductions were recorded in most trials when probiotics were used adjunctively to scaling and root planing (SRP) [18]. In a recent systematic review, *L. reuteri* showed interesting benefits as an adjunct to non-surgical periodontal treatments [19].

Martin-Cabezas et al.’s meta-analysis incorporated four RCTs, three of which were included in the quantitative synthesis [20]. All analyzed studies evaluated the effects of *L. reuteri* and recorded a statistically significant gain in clinical attachment level (CAL) and a reduction in BoP, while subgroup analyses showed significant PPD improvements in moderate and deep pockets, respectively [20]. To conclude, certain probiotic strains appear to influence the composition of subgingival microbiota, potentially creating a less pathogenic environment through modulation of host immune responses [21]. Hence, the aim of the present review was to evaluate the efficacy of probiotics as an adjunct to NSPT.

## 2. Materials and Methods

An electronic search was conducted on PubMed, Scopus, Medline, and Google Scholar for published RCTs written in English in order to retrieve all the available literature. The following search keywords were used: probiotics, periodontal therapy, adjunctive therapy, and non-surgical periodontal therapy.

Studies were included in this literature review in accordance with the following inclusion criteria: (1) studies focused on the clinical effects of probiotics as an adjunct to NSPT; (2) studies performed in vivo on humans; and (3) randomized controlled trials (RCTs).

Studies were excluded from this review in accordance with the following exclusion criteria: (1) studies that did not use probiotics as an adjuvant to NSPT or used it to treat other oral conditions and (2) studies not available in English. The mean age of participants ranged from 35 to 55 years. The majority of studies involved middle-aged adults affected by periodontitis and had relatively homogeneous populations in terms of age and periodontal susceptibility.

### 2.1. Study Selection

**Figure 1:**
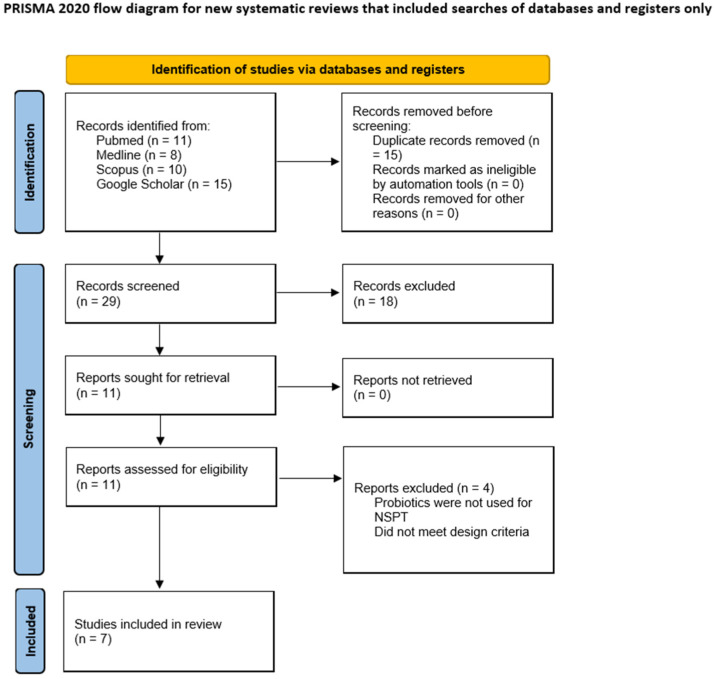
Flowchart article retrieval process.

A reviewer (A.C.) independently assessed articles based on inclusion criteria by title and abstract. After this initial screening stage, the full texts of articles that met these criteria were obtained. Subsequently, a full text examination of the selected studies was undertaken. Clinical data (i.e., PD and CAL) were extracted and reported as means and standard deviations. The article selection process is illustrated in Figure 1.

### 2.2. Charting Data

Clinical data were extracted and will be reported in a table. The following data were recorded:

- Authors
- Study design
- Setting
- No. of patients
- Gender
- Mean age
- Smoking habit
- Type of periodontitis
- Treatment
- Results
- Follow-up

## 3. Results

### 3.1. Probiotics’ Effects on Human Health

Probiotics seem to promote several beneficial effects on human health with regard to both the immune system and oxidative stress. Oxidative stress occurs when there is an imbalance between prooxidants and antioxidants, leading to cellular damage through peroxidation and protein denaturation hydroxylation [22,23]. Probiotics showed potential in mitigating this damage through enzymatic and non-enzymatic mechanisms. They are capable of producing antioxidant enzymes such as superoxide dismutase (SOD), glutathione peroxidase (GPx), and catalase (CAT), which neutralize reactive oxygen species (ROS). Additionally, probiotics can increase levels of non-enzymatic antioxidants like glutathione and vitamins C and E. Therefore, probiotics administration has beneficial effects on several inflammatory diseases, such as inflammatory bowel diseases (IBDs), metabolic disorders such as obesity, and even diabetes, regulating insulin resistance [23]. In fact, probiotics are able to regulate cytokine production by increasing IL-10 and TGF-β, which promotes regulatory T cell (Treg) function, and by reducing TNF-α and IL-6, which helps mitigate inflammatory conditions [24]. They also have a neuroprotective effect, modulating neurotransmitter production in Alzheimer’s disease and Parkinson’s disease. And lastly, a reduction in the level of cholesterol has been reported thanks to the improvement of endothelial function, lowering the risk of atherosclerosis [23]. Specific species of bacteria are associated with different effects: *Lactobacillus rhamnosus* protects against exercise-induced oxidative stress [25], *Bifidobacterium animalis* removes hydroxyl radicals and superoxide anions in diabetic patients [26], and *Lactobacillus fermentum* produces Mn-SOD, reducing inflammation and enhancing the health of the gastrointestinal system [27].

### 3.2. Probiotics’ Effects on Oral Health

Oral diseases are often caused by a disrupted oral microbiota, where a shift occurs between protective bacteria and pathogens species such as *Streptococcus mutans*, triggering an inflammatory cascade leading to tissue destruction. Probiotic species, particularly *Lactobacillus* and *Bifidobacterium*, are known for their ability to adhere to oral surfaces, thereby competing for adhesion sites and nutrients with harmful bacteria. Moreover, these beneficial microorganisms produce antimicrobial compounds like bacteriocins and organic acids, which help inhibit periodontal pathogens [28]. The rationale behind the use of probiotics in periodontal health is associated with biofilm-inducted periodontal inflammation. In fact, periodontitis onset has been reported in cases where a susceptible host recorded a variation in oral microflora marked by a reduction in so called “beneficial bacteria” and an increase in periodontopathogen bacterial species (e.g., *Aggregatibacter actinomycetemcomitans, Porphiromonas gingivalis*, and *Treponema denticola*) [29]. Probiotics are able to modify the pH of saliva, thereby preventing plaque formation and producing antioxidants that use free electrons, which prevent bacterial adhesion [28]. A recent review highlighted how probiotic interventions affect periodontal health outcomes. The results indicate that administering specific probiotic species, including various *Lactobacilli* and *Bifidobacteria*, leads to a significant reduction in the colony-forming units (CFU) of periodontal pathogens [30]. Studies report improvements in clinical parameters such as reduced gingival bleeding and lower inflammation scores, suggesting that probiotics can modulate the local inflammatory response in periodontal tissues [30,31].

### 3.3. Probiotics in Non-Surgical Periodontal Therapy

**Figure 2:**
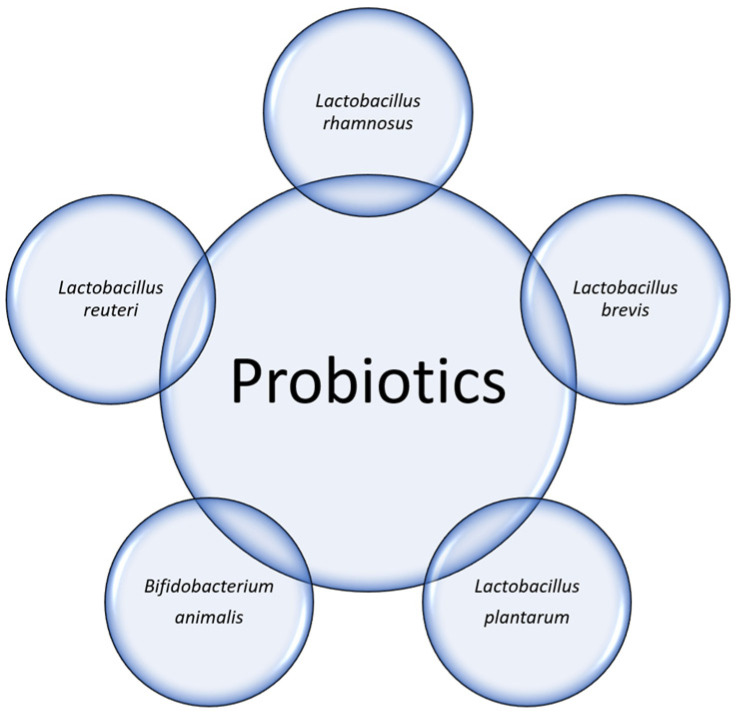
Probiotics species analyzed.

**Figure 3:**
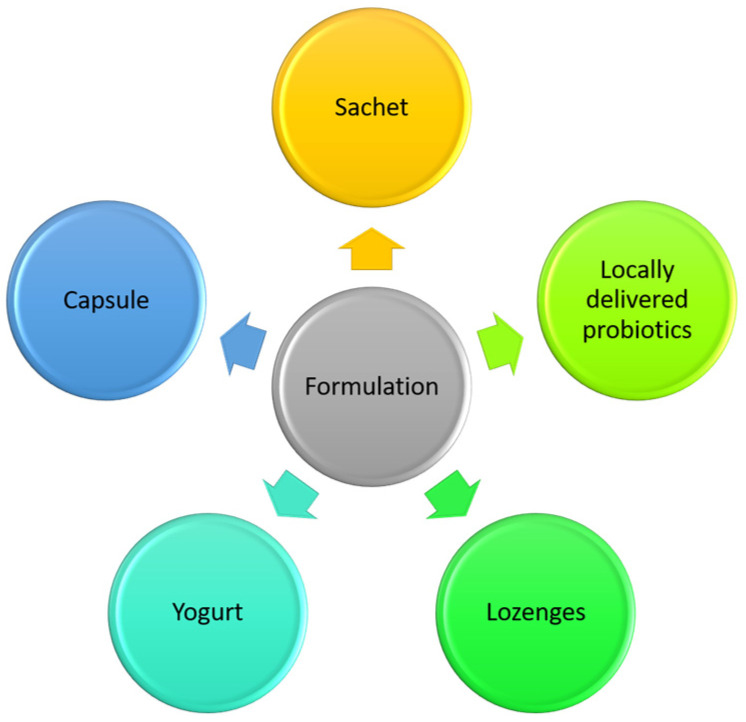
Probiotics formulations.

Eleven articles were found from the electronic search and four of these were excluded after title analysis; therefore, seven articles were included for revision. NSPT is generally effective for the treatment of most periodontal cases. Surgery is recommended only when a residual periodontal probing depth ≥ 6 mm is recorded [9]. In order to avoid surgery and to enhance NSPT, the effects of probiotics on periodontal health were evaluated [18]. The characteristics of the analyzed studies and clinical findings are presented in Table 1. Statistically significant differences were found by İnce et al., who assessed the effects of *Lactobacillus reuteri* lozenges administrated twice a day for three weeks as an adjunct after NSPT and recorded PD reductions of 1.70 ± 0.31 mm and 0.55 ± 0.26 mm between test and control groups, respectively, at one year of follow-up [32]. Similarly, Minic et al. [33] highlighted PD reductions of 4.08 ± 0.22 mm and 4.72 ± 0.36 mm between test and control groups, respectively, at one month of follow-up using as an adjunct to NSPT an application of a mixture of probiotic bacteria to the periodontal pockets. This mixture contained 6.5 billion live *Lactobacillus acidophilus* with a concentration of 107 unit-forming colonies (CFU), at least 107 CFU of *Bifidobacterium infantis*, and at least 106 CFU of *Enterococcus faecium*. Topical application of the probiotic mixture was performed daily for a period of 5 days [33]. In the same way, Sachelarie et al. recorded PD reductions of 3.11 mm and 5.30 mm between test and control groups, respectively; the test group received a daily capsule containing *Lactobacillus reuteri* (2 × 109 CFU) for 8 weeks while the control group received a placebo [34]. Conversely, Morales et al. investigated the effects of *Lactobacillus rhamnosus* (2 × 107 colony-forming units (CFU)/day) after NSPT. Patients were instructed to take one probiotic sachet/day (150 g) administered for 3 months, but no statistically significant differences were recorded in terms of PD between test and control groups at one year (PD test: 2.1 ± 0.5 mm; PD control: 2.0 ± 0.2 mm) [35]. Pelekos et al. [31] similarly recorded no significative difference in terms of PD at six months follow-up (PD test: 2.6 ± 0.4 mm; PD control: 2.9 ± 0.6 mm); in this case the test group received probiotics lozenges (*L. reuteri*, 108 CFU) and patients were instructed to take the lozenges twice per day for 28 days. Likewise, Ozener et al. recorded PDs of 2.05 ± 0.36 mm and 2.06 ± 0.35 mm between test and control groups, respectively, at three months follow-up; however, in this case probiotics were administered by yogurt [36]. And lastly, Pudgar et al. [37] administrated a gel prepared by mixing water and probiotics/placebos in all periodontal pockets deeper than 4 mm using a specifically designed applicator. Additionally each patient received three boxes containing 30 probiotic/placebo lozenges and was instructed to take one per day for 3 consecutive months. The probiotic gel contained 6.0 × 10^9^ CFU/mL of *L. brevis* and *L. plantarum,* while the probiotic lozenges contained 1.2 × 10^9^ CFU/mL of each bacterium; in that study, at three months the PD median value for the test group was 3.0 mm and for the control group it was 3.1 mm [37]. All results are summarized in Table 1. The probiotics species analyzed and their main formulations are summarized in Figure 2 and Figure 3, respectively.

## 4. Discussion

The aim of the present review was to provide clinicians with a guideline regarding whether probiotics may be used as an adjunctive therapy to NSPT. This topic was investigated due to the potential of probiotics to modulate both oral microbiota and inflammatory responses [28]. In particular, it seems that only particular genera of bacteria (e.g., *Lactobacillus* and *Bifidobacteria*) are capable of competing with periodontopathogens showing antimicrobic properties, and yet clinical outcomes were inconsistent across studies [27,32,35,37]. Some of the analyzed studies showed significant results in term of PD reduction and CAL gain, such as those by İnce et al. and Minic et al.; however, their results are difficult to compare. In the first study, probiotics were administrated as lozenges, while in the second study a mixture was applied in periodontal pockets. İnce et al. obtained a PD reduction of only about 1.5 mm compared to the 0.5 mm obtained by Minic, suggesting that prolonged probiotics administration delivered in lozenges is more effective than a locally delivered formulation. However, the poor results obtained by Minic could be associated with the short follow-up period of 1 month [8,32,33]. The best results were obtained by Sachelarie et al., who recorded for their test group a PD reduction of about 2.4 mm compared to the 0.4 mm reduction in the control group; similar to İnce et al., the probiotic was administrated in a capsule for a period of 2 months [34]. These promising results should be assessed carefully for potential biases, as a two-month follow-up is relatively short; it was not disclosed who performed the re-evaluation of the patients or if the examiner was blinded to the allocation; and finally, because differences in oral hygiene levels between test and control groups were not evaluated.

A comprehensive network meta-analysis including 33 RCTs up to November 2023 confirmed that *Lactobacillus,* especially *L. reuteri*, showed the best improvements in probing pocket depth (PPD), clinical attachment level (CAL), and bleeding on probing (BoP) compared to other probiotics [38]. Subgroup analysis indicated that lozenges or capsules administered twice a day for over one to three months provided encouraging benefits [39].

This meta-analysis also highlighted that while short- and medium-term benefits were evident, probiotic effects tend to reduce after 6–12 months, underlining the need for extended-duration research to evaluate sustained efficacy and inform more robust clinical practices [38].

A 2023 RCT conducted in smokers with Stage III periodontitis demonstrated that *L. reuteri* lozenges showed comparable outcomes in reducing PPD and gingival inflammation when compared to systemic antibiotics, although no significant differences were observed in CAL. This suggests probiotics may represent an alternative to antibiotics in selected populations [40]. Recently a study focused attention on the role of *Limosilactobacillus reuteri* in modulating the subgingival microbiota and reducing periodontal inflammation. Specifically, *L. reuteri* significantly lowered levels of periodontopathogenic species such as *P. gingivalis* and *T. forsythia* while promoting a healthier microbial profile. These microbiological shifts could be associated with clinical improvements in periodontal clinical parameters, enhancing the potential of *L. reuteri* as an adjunct to periodontal therapy [41].

Furthermore, a recent systematic review included 24 randomized clinical trials in which probiotics were evaluated as adjuncts to periodontal therapy for gingivitis and periodontitis. The findings indicated that probiotics significantly reduced the plaque index and bleeding on probing in periodontitis, while results for gingivitis were non-significant. However, the review highlighted the importance between statistical significance and clinical relevance, stressing the concept that treatment effects should be always interpreted in real-world dental practice [42].

The remaining analyzed studies did not show any significative results in terms of PD reduction, while some of them recorded a reduction in gingival inflammation with a follow-up period that ranged from 3 months to 1 year, showing no effects in either the short or long term [36,37]. These results are in line with the literature. In fact, a recent literature review highlighted that probiotics have limited or modest effects in terms of clinical outcomes and reported a mean periodontal probing reduction of 0.5 mm; in addition, the best results were obtained with a preparation containing *L. ramnosus* and *L. reuteri* [43].

Despite the limitations of the present study, such as the heterogeneity of included studies differing in probiotic species, dosages, delivery methods, and follow-up durations, we may conclude that probiotics represent an adjunctive approach to NSPT. However, their clinical benefits remain questionable due to both the lack of a well-defined protocol and the absence of guidelines concerning the best formulation and application. Therefore, further research is needed to determine both the most effective probiotic and the most reliable treatment. Actually, probiotics could be considered as an adjunct to periodontal therapy, but their use should be tailored to each individual patient’s needs and clinical scenarios.

## 5. Limitations

This review was limited by the small number of randomized controlled trials (RCTs) available on the adjunctive use of probiotics in non-surgical periodontal therapy (NSPT). The included studies showed a high degree of heterogeneity in terms of probiotic species, delivery methods, dosages, and follow-up durations, which made it difficult to compare outcomes. Furthermore, the different follow-up periods and lack of an eventual oral hygiene protocol could represent a source of bias for the present study.

## 6. Future Directions

Future research should focus on multicenter RCTs with standardized protocols, probiotic formulations, dosages, and treatment durations. Additionally, exploring patient-specific factors, such as baseline microbiota composition, may be useful to establish proper probiotic therapies in periodontal care.

## 7. Conclusions

This narrative review evaluated the efficacy of probiotics as an adjunct to non-surgical periodontal therapy (NSPT). While certain probiotic genera, such as *Lactobacillus* and *Bifidobacterium*, seem to show potential in modulating oral microbiota and reducing inflammation, the overall clinical benefits remain inconsistent due to variability in study protocols, probiotic formulations, and follow-up durations. More RCTs are needed to establish clear guidelines for probiotic use in periodontal therapy.

## Figures and Tables

**Table 1:** Characteristics and clinical findings of analysed studies.

Authors	Study Design	Setting	No. of Patients	Gender (Male/Female)	Mean Age (Years)	Smoking Habit	Type of Periodontitis	Treatment	Results (Main Outcome of the Analysed Study)	Follow-Up
İnce, G. et al. (2015) [32]	RCT	University	30	17 M/13 F	Test Group 41 ± 3.17 Control group 42.20 ± 2.78	Smokers were excluded	Chronic Periodontitis	Test Group NSPT + probiotics lozenges (*Lactobacillus reuteri*) Control group NSPT + placebo	Test Group PD (mm) Baseline: 5.85 ± 0.54 1 year: 1.70 ± 0.31 Control group PD (mm) Baseline: 5.57 ± 0.39 1 year: 0.55 ± 0.26	1 year
Morales A. et al. (2016) [35]	RCT	University	28	14 M/14 F	52.7 ± 7.3	22 N.S./6 S	Chronic Periodontitis	Test Group NSPT + probiotics sachet (*Lactobacillus rhamnosus*) Control group NSPT + placebo	Test Group PD (mm) Baseline: 2.7 ± 0.6 1 year: 2.1 ± 0.5 Control group PD (mm) Baseline: 2.5 ± 0.3 1 year: 2.0 ± 0.2	1 year
Pelekos G. et al. (2019) [31]	RCT	University	59	20 M/39 F	54.1 ± 29.0	Smokers were excluded	Chronic Periodontitis	Test Group NSPT + probiotics lozenges (*Lactobacillus reuteri*) Control group NSPT	Test Group CAL level (mm) Baseline: 4.2 ± 1.3 6 months: 4.0 ± 1.3 PD (mm) Baseline: 3.1 ± 0.6 6 months: 2.6 ± 0.4 Control group CAL level (mm) Baseline: 4.9 ± 1.7 6 months: 4.6 ± 1.6 PD (mm) Baseline: 3.5 ± 1.0 6 months: 2.9 ± 0.6	6 months
Pudgar P. et al. (2020) [37]	RCT	University	40	18 M/22 F	Test Group 45.9 ± 8.0 Control group 46.7 ± 11.0	27 N.S./13 S	Advanced Periodontitis (Stage III–IV)	Test Group NSPT + probiotics lozenges plus locally delivered probiotics (*Lactobacillus brevis* and *Lactobacillus* *Plantarum*) Control group NSPT + placebo	Test Group CAL level mm (median) Baseline: 4.3 (3.8; 4.9) 3 months: 3.6 (3.1; 4.2) PD (mm) Baseline: 3.9 (3.7; 4.2) 3 months: 3.0 (2.9; 3.2) Control group CAL level mm (median) Baseline: 4.5 (4.0; 5.9) 3 months: 3.7 (3.3; 4.9) PD (mm) Baseline: 4.0 (3.6; 4.3) 3 months: 3.1 (2.8; 3.3)	3 months
Minic I. et al. (2022) [33]	RCT	University	80	N.A.	35–55	N.A.	Periodontitis (PD ≥ 5mm)	Test Group NSPT + locally delivered probiotics Control group NSPT	Test Group PD (mm): Baseline: 5.30 ± 0.46 1 month: 4.08 ± 0.22 Control group PD (mm) Baseline: 5.22 ± 0.56 1 month: 4.72 ± 0.36	1 month
Ozener H. et al. (2023) [36]	RCT	University	30	15 M/15 F	Test Group 41.40 ± 6.8 Control group 42.27 ± 8.8	Smokers were excluded	Stage III Grade B	Test Group NSPT + *Bifidobacterium animalis* (yogurt) Control group NSPT + placebo	Test Group PD (mm) Baseline: 2.76 ± 0.38 3 months: 2.05 ± 0.36 Control group PD (mm) Baseline: 2.59 ± 0.43 3 months: 2.06 ± 0.35	3 months
Sachelarie L. et al. (2025) [34]	RCT	University	80	30 M/50 F	Test group 45 ± 6 Control group 46 ± 5	N.A.	Moderate/Severe periodontitis	Test Group NSPT + *Lactobacillus reuteri* (capsule) Control group NSPT + placebo	Test Group PD (mm): Baseline: 5.53 2 months: 3.11 GI Baseline: 2.70 2 months: 0.87 Control group PD (mm): Baseline: 5.71 2 months: 5.30 GI Baseline: 2.42 2 months:1.58	2 months

RCT: randomized controlled trial; NSPT: non-surgical periodontal therapy; PD: probing depth; CAL: clinical attachment level; GI: gingival index; S: smoker; N.S.: non-smoker; NSPT: non-surgical periodontal therapy.

## Data Availability

All data are available and published online.

## Abbreviations

The following abbreviations are used in this manuscript:

NSPT	Non-surgical periodontal therapy
SRP	Scaling and root planing
PD	Probing depth
CAL	Clinical attachment level
BoP	Bleeding on probing
EFP	European Federation of Periodontology
SPC	Supportive periodontal care
MINST	Minimally invasive non-surgical technique
SOD	Superoxide dismutase
GPx	Glutathione peroxidase
CAT	Catalase
ROS	Reactive oxygen species
IBDs	Inflammatory bowel diseases
CFU	Colony-forming units
